# Carcass Quality, Meat Quality and Sensory Properties of the Dual-Purpose Chicken Lohmann Dual

**DOI:** 10.3390/foods7100156

**Published:** 2018-09-25

**Authors:** Lisa Siekmann, Lisa Meier-Dinkel, Sabine Janisch, Brianne Altmann, Claudia Kaltwasser, Christian Sürie, Carsten Krischek

**Affiliations:** 1 Department of Animal Sciences, Division for Quality of Animal Products, Georg-August-University Goettingen, D-37075 Goettingen, Germany; lisa.siekmann@agr.uni-goettingen.de (L.S.); sabine.janisch@agr.uni-goettingen.de (S.J.); brianne.altmann@agr.uni-goettingen.de (B.A.); ckaltwa@gwdg.de (C.K.); 2 Institute of Food Quality and Food Safety, Foundation University of Veterinary Medicine, D-30173 Hannover, Germany; carsten.krischek@tiho-hannover.de; 3 isi GmbH, D-37124 Rosdorf/Goettingen, Germany; 4 Farm for Education and Research Ruthe, Foundation University of Veterinary Medicine Hannover, D-31157 Ruthe/Sarstedt, Germany; christian.suerie@tiho-hannover.de

**Keywords:** production performance, descriptive sensory analysis, MORS-blade, animal welfare

## Abstract

Over 40 million day-old layer line cockerels are culled in Germany each year, due to economic reasons, leading to a recently instigated research focus on the potential of dual-purpose breeds as an alternative to conventional poultry husbandry, especially the practice of culling. This paper aims to explore and assess the dual-purpose chicken breed “Lohmann Dual” (LD) performance (*n* = 30) and sensory characteristics (*n* = 48). Carcass and meat quality traits are evaluated, and descriptive sensory analysis of breast muscles is conducted. To define the scope of characteristics, a market sample of “Ross” Line (*n* = 35) is adducted. LD carcasses are characterized by higher leg than breast yield; carcass, breast and leg weights are higher in Ross. LD meat has a lower pH, differs in color, has higher drip and thawing losses, but lower cooking loss. LD breast muscles are firmer as indicated by shear force measurements, which is confirmed through the sensory analysis. Appearance, odor and flavor differ between the lines. Overall, distinguishable differences are found between both breeds. Further research should focus on the marketing aspect of the dual-purpose line, as some characteristics could draw consumers to this product. Animal welfare and ethical concerns should further be considered when considering dual-purpose breeds as a feasible alternative to culling.

## 1. Introduction

One-day-old male layers are usually culled directly after hatching by homogenization, as rearing these animals would be economically detrimental, due to their inefficient growth rate [1,2]. Investigations into the rearing of male layers showed that their performance is less attractive for meat production [3,4,5]. According to the animal welfare regulations of the European Union and Germany it is forbidden to cause pain, suffering or harm to animals without a sensible reason. As the culling of millions of chicks, due to economic reasons is not clearly justifiable as a “sensible reason”, this subject has become the topic of much criticism by animal welfare organizations, the public, and policy-makers. Nonetheless, attempts to forbid the killing of chicks have not yet been successful, mainly because suitable alternatives are not yet available or economically relevant. Unfortunately, insemination with sex-sorted sperm, a method for sex predefinition of the offspring in mammalians, is not possible in birds, because the egg cell (female gamete) determines the sex of the chicks [6]. However, there are other alternatives, like sex determination in ovo with the removal of the male eggs before hatching or the rearing of the layer cockerels using chicken genetics bred for dual-purpose use (meat, egg) and these will be briefly discussed in the following paragraphs.

Sex determination in ovo with near-infrared Raman spectroscopy enables contactless differentiation at day 4 of egg incubation via analysis of circulation blood spectra. According to Galli et al. [7] the method is accompanied with lower hatching rates, requires egg-windowing, and the sexing is only 90% accurate; therefore a higher amount of eggs would have to be incubated. However, technique improvements could increase the hatching rate and accuracy [7]. Sex determination via analysis of estradiol and estrone sulfate in the allantoic fluid at day 9 of incubation [8] might also be an alternative. Up until now this method has several disadvantages, due to the invasive technique for collection of the allantoic fluid, i.e., the risk of cross-contamination between the eggs, long duration of analysis times, reduced hatching rates and the possibility of an embryonic pain sensation during the procedure [9]. Sex differentiation by hyperspectral image analysis is also an interesting non-invasive method, but it is limited to genetics with dimorphism in down color, and it only has a determination accuracy of 85% starting at day 13 of incubation [10].

The rearing of dual-purpose breeds is perhaps the most favorable alternative, at the moment, until the disadvantages of sex determination in ovo are resolved. However, this alternative is still hindered by drawbacks. Due to the negative correlation between egg-production and body conformation traits [11], dual-purpose chickens do not have the same performance abilities compared to highly specialized layer or broiler genetics. The dual-purpose lines showed, depending on the use for meat or egg production, higher feed consumption rates, lower growth properties and breast yields, as well as lower egg production compared to the specialized breeds [12,13]. Although these disadvantages of the slow-growing dual-purpose genetics may seem unfavorable, investigations show that selection for faster-growing has led to an impaired flavor of the meat [14], which could be reinstated through the use of breeds with reduced growing properties, like dual-purpose breeds. The dual-purpose breed “Lohmann Dual” (LD) is explicitly bred for simultaneous use of cockerels in meat, and hens in egg production. This enables the switch from highly specialized hybrids, where usually one sex is favored for production, to a well-balanced (slow-growing) dual-purpose breed, where both sexes are utilized, resulting benefits to animal welfare.

In the present study, the carcass and meat quality traits and especially the sensory properties of the commercial dual-purpose line LD were evaluated. To provide insights into the potential advantages of this genetic, market samples of the frequently slaughtered fast-growing genetic Ross 308 were also analyzed as a point of comparison. As far as we know, only a few studies have been published concerning the objective sensory characteristics of slow-growing birds in general [15] and dual-purpose chicken in particular, but other studies focusing on sensory attributes of poultry meat are available [16,17]. The aim of the study was to provide a backdrop for further research into understanding differing sensory profiles based on poultry breed, as well as a better understanding into the meat quality aspects of dual-purpose breeds, particularly LD. Moreover, characteristics of LD offer fundamental information for assessment of market potential and determination of future customer groups.

## 2. Materials and Methods 

### 2.1. Ethical Declaration

This study was conducted in compliance with the German and European animal welfare regulations for animal husbandry, transport and slaughter.

### 2.2. Materials

#### Animals and Sample Collection

Chickens of the dual-purpose breed Lohmann Dual (LD, cockerels, Lohmann Tierzucht GmbH, Cuxhaven, Germany) were raised under standardized conditions at the Farm for Education and Research Ruthe (Foundation University for Veterinary Medicine Hannover). The feeding program was divided in commercial starter- (12.6MJ ME, 22% crude protein, non genetically modified organisms (GMO), day 0–10), two slow-growth-rearing-ratios (grower I: 12.2MJ ME, 19% crude protein, non GMO, day 11–38; grower II: 12.6MJ ME, 19% crude protein, non GMO, day 39–50) and a finisher ratio (13.2MJ ME, 19,5% crude protein, non GMO, day 51–63). Feed was acquired by Mega Tierernährung GmbH and Co. KG (Haldensleben, Germany). Feed and water were offered ad libitum. Feed was withdrawn 10 h before slaughter. The birds were reared with a stocking rate below 25 kg/m^2^ on, sieved wood shavings (600 g/m^2^) litter and had straw bundles as material for perching and investigation. The barn was continuously illuminated in the first 48 h, with 4 h darkness at day 3 and 16 h light and 8 h dark from day 4 until slaughter. At cooping, temperature was about 33 °C ambient temperature. Temperature decreased continuously to 18.8 °C at day 63, with a maximal humidity of 80%.

Chicken carcasses from LD (*n* = 78, age: 64 days,) were randomly selected after commercial slaughter. The LD birds were electrically stunned in a water bath (9 s, 100–150 mA), exsanguinated by neck cut, scalded (58 °C, 150 to 210 s), eviscerated, and chilled at 4 °C for at least two hours. Whole carcasses of Ross 308 birds (Aviagen UK Ltd., Midlothian, UK) with a mean age of 42 days were acquired from a commercial abattoir. These animals were stunned with CO_2_ in two phases (1. phase: 23% O_2_, 27% CO_2_ for 1 min; 2. phase: 70% CO_2_, 0% O_2_ for 2 min). They were exsanguinated after neck-cutting, scalded (54 °C, 210 s), eviscerated, pre-chilled for one hour at 0 °C and subsequently chilled for further two hours at 0.5 °C.

After chilling, all carcasses were transported at 4 °C for ca. 4 h to the laboratory facilities of the Department of Animal Sciences of the Georg-August-University. The following procedure was equally conducted for both breeds: Twenty-four hours after slaughter (24 h postmortem [p.m.]) the carcasses were weighed and manually dissected. 30 *Musculi pectorales superficiales* (MPS) were used for analysis of the meat quality traits. Due to different sizes of the breast muscles a total of 48 MPS of LD and 24 MPS of Ross were prepared for sensory evaluation, and any remaining breast muscles were appointed for training panelists (definition of sensory attributes and the evaluation scheme, training of scale usage). The samples for sensory analysis were sealed in an air-evacuated polyethylene bags and frozen at −20 °C until training or evaluation.

### 2.3. Methods

#### 2.3.1. Carcass-Characteristics and Meat Quality Traits

MPS (boneless, skinless) and both legs (with bones, skinless) were weighed. The MPS and leg yields in percent were calculated in relation to the carcass weight.

The pH values were determined 24 h p.m. with a pH-meter by insertion of the pH-electrode and a thermometer (Knick, Portamess 913, Berlin, Germany) into the center of the MPS. Beforehand, the pH-meter was calibrated using standardized buffers (pH 4.0, 7.0).

Lightness (L*), redness (a*) and yellowness (b*) values were determined with a chromameter CR-400 (KONICA MINOLTA, Langenhagen, Germany) in triplicate on the ventral non-defected (no discolorations or petechial bruises) surface of the MPS. The aperture size was 8 mm, the illuminant D65 and standard observer angle was 10°.

To determine drip loss (DL), the intact MPS were hung in separate boxes equipped with a lid and stored at 4 °C between 24 h and 72 h p.m. The weights of the samples were determined 24 h and 72 h p.m. and the percentage of drip loss was calculated ((Weight_24 h p.m._ − Weight_72 h p.m._/Weight_24 h p.m._) × 100) [18]. All drip loss samples were then vacuum packaged in plastic bags and frozen at −20 °C until thawing/cooking loss and shear force analysis was conducted.

**Figure 1:**
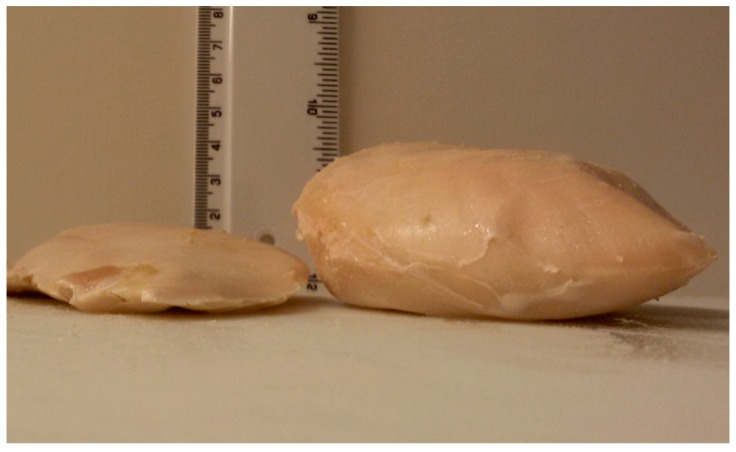
Picture of *Mm. pectorales superficiales*, collected from the Lohmann Dual (LD) at day 64 (left) and Ross at day 43 (right), after cooking for sensory analysis.

Three months after slaughter the packaged drip loss samples were thawed overnight at 4 °C, which was followed by an acclimation at room temperature for about one hour. The MPS were unpacked, carefully dried with paper and reweighed to determine the percentage thawing loss (TL) [18]. The thawed MPS were packaged again and cooked in a hot water bath (3043, Köttermann, Uetze/Hänigsen, Germany) at 77 °C for approximately 30 min (LD) and 60 min (Ross) to reach a core-temperature between 74 and 75 °C. The core-temperature of each sample was determined by insertion of a temperature electrode into the center of the MPS (testo 926, Lenzkirch, Germany). After cooking (Figure 1), the samples were, unpacked, carefully dried with paper, stored at room temperature and reweighed for the analysis of the percentage of cooking loss (CL) [18].

For the determination of shear force (SF) and shear energy (SE) values, the samples were measured using a TA.XTplus texture analyser (Stable Micro Systems Ltd., Surrey, UK), with a 5 kg load cell and a Meullenet-Owens Razor Shear Blade (MORS-Blade). The conditions for the shear analysis according to Xiong et al. [19], were as follows: Pre-test-speed 100 mm/min, test speed 600 mm/min, post-test-speed 300 mm/min, trigger type 10 g (contact force), blade penetration depth 20 mm and data acquisition rate 200 PPS. Each intact MPS-sample was penetrated six times perpendicular to muscle fibre orientation. The following results were measured: SF (in N) was fixed as the first turning point in the deformation curve representing the maximum force to cut the first muscle fibres. SE (in N × mm) represented the whole area under the curve from the start to the turning point of the SF-measurement.

#### 2.3.2. Descriptive Sensory Analysis

Descriptive analysis was selected for objective sensory evaluation of the samples [20,21]. Evaluation was performed by ten trained panelists, who were experienced in descriptive sensory profiling of pork and poultry products. The sensory panel was selected according to ISO 8586-1 [22]. Training with chicken samples lasted for 7 weeks (2 days/week, duration: 1.5 to 2 h per session) according to Lawless [21]. The final sensory evaluation was performed over two weeks (2 days/week, duration: 1.5 to 2 h per session). All panelists evaluated samples of both breeds in triplicate in one session. Each sample was evaluated for up to 12 min, followed by a break to neutralize the senses of smell and taste with water and white bread. Sensory training and evaluation were carried out in the Laboratory for Sensory Analysis and Consumer Research at the University of Goettingen equipped—according to ISO 8589:2010 [23]—with ten individual cubicles, air exchange and standardized lighting. During the sensory training and evaluation phases the panelists were in the cubicles and evaluated each sensory attribute on a 10 cm unstructured line scale with scale endpoints as given in Table 1, but mainly ranging from 0 (weakest perception) to 100 (strongest perception) [24]. The software EyeQuestion (Logic8 BV, Elst, The Netherlands) was used for data acquisition.

**Figure 2:**
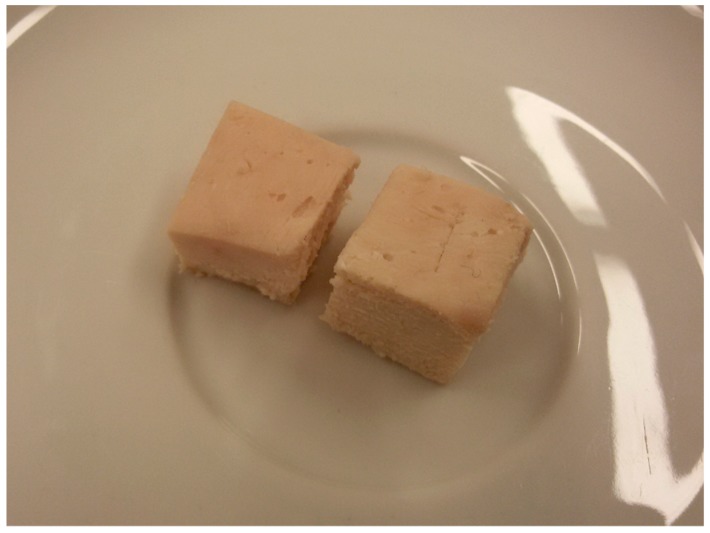
Picture of a sample, prepared for sensory evaluation.

The samples for training and objective sensory evaluation were prepared as presented above for cooking loss. Due to organization of training and evaluation sessions, the samples have been stored for 6 weeks to 3 months. Therefore, determination of SF and SE was performed parallel to final sensory evaluation after 3 months. The muscles were cut into pieces (10 mm × 10 mm × 10 mm, Figure 2) and were stored for up to 2 min on a heated plate until evaluation. For sensory analysis the samples were allocated in a randomized order, labeled with a 3-digit random code, and given to each single panelist for evaluation. Due to the clear weight differences of the LD and Ross MPS, either 2–3 (LD) or 5 (Ross) samples were obtained from each muscle. Each panelist evaluated two pieces per sample. The results of 120 assessments per poultry genetic were used for further statistical analysis. Detailed information about the sensory attributes evaluated during the sessions is presented in Table 1. The attribute list was permanently available for each panelist during each evaluation session.

### 2.4. Chemicals

Caffeine (PubChem CID: 2519), Citric acid monohydrate (PubChem CID: 22230) and Saccharose (PubChem CID: 5988) for the sensory training were obtained from Carl Roth (Karlsruhe, Germany). Standard solutions for pH calibration (pH 4.0 and 7.0) were obtained from Sigma-Aldrich Chemie GmbH (Taufkirchen, Germany).

### 2.5. Data Analysis

Data analysis was performed with Statistical Analysis System (SAS, version 9.3., 2011, SAS Institute Inc., Cary, NC, USA). After analysis of the carcass-characteristics and meat quality values for normality (Shapiro-Wilks-test) parameters showing normal distribution were analyzed using a 2-sample-t-test. Pooled values were used for variance equality and Satterthwaite approximation for unequal variances. The other (non-parametrical) data were analyzed with the Wilcoxon-two-sample test. ANOVA (mixed model) was applied for statistical analysis of the results from the sensory evaluation using the GLM procedure of SAS. The statistical model contains genetic of the animals (Ross; LD) as a fixed effect and additionally the panelist and day of evaluation as random effects. An alpha-level of 0.05 is considered significant in all statistical tests.

For reasons of clarity the mean and standard deviation values of the data (parametrical and non-parametrical) are presented in the tables and the non-parametrical data are marked with a * in the appropriate tables.

## 3. Results and Discussion

### 3.1. Carcass-Characteristics and Meat Quality Traits

In the present study, the carcass, MPS, and leg weights, as well as the MPS yields of the LD animals were lower (*p* < 0.0001 for all) compared to the Ross broiler. Only the leg yields of the LD birds were higher (*p* < 0.0001) than the results of the Ross birds (Table 2). Performance of LD in this study was similar to recently published data of 63 day-old LD with regard to carcass and leg weights [25]. However, considering a dressing percentage of 67% [26], carcass weights in the present study were clearly higher compared to weight results of 63 day-old LD presented by Habig et al. [27]. The carcass, breast and leg weights, as well as breast and leg yield values of the Ross broiler were mainly in line with other investigations [28,29]. Although some comparisons of meat quality traits for fast- and slow-growing broiler exist, comparable studies are rare, due to differences in chicken breed, age and handling procedure. However, in accordance to the present results, lower carcass and breast weights in combination with higher leg yields were also found for LD birds by Mueller et al. [26] and in general for slow-growing birds in other publications [25,30,31,32,33]. Murawska and Bochno [5] found increasing leg yields with increasing age in (slower-growing) layer lines, but decreasing leg yields in Ross after three weeks of age. Therefore, a suggestion would be to market animals of the LD genetic as a whole bird. There might be a chance for slow-growing genotypes in Europe, as the Label Rouge program in France markets poultry products [25,33], which are mainly consumed as whole carcasses [34]. On the contrary, Castellini et al. [14] considered industries demand for processed poultry products as a limiting factor for the marketing of whole carcasses, which have mainly been sourced from free-range or organic production systems.

With regard to the meat quality traits (Table 3), the MPS of the Ross genetic had higher pH_24 h p.m._ values (*p* = 0.0079) than the LD animals. These results are in agreement with other studies that present lower pH values in the MPS of LD and other slow-growing birds [26,35,36,37,38]. Possible reasons for these pH differences are higher stress sensitivity accompanied with a higher activity during transport and slaughter (e.g., wing-flapping, struggling) of slow-growing birds [36,38,39].

The Ross L*_24 h p.m._ and b*_24 h p.m._ values were higher, i.e., lighter and more yellow (*p* < 0.0001 for both), and the a*_24 h p.m._ values lower (*p* < 0.0001), i.e., less red in comparison to the LD animals (Table 2). Hemoglobin, as well as myoglobin content, and the different redox forms of the myoglobin, are the main determining factors of meat color [40] depending on factors like meat species, age or muscle type [17]. Investigation by Berri et al. [41] also showed higher L* and lower a* values in selected birds with higher muscle mass, as is the case in this study, where Ross animals have larger MPS compared to the LD animals. This result was also supported by the investigations of Zhao et al. [42]. However, the age difference between Ross and LD should be considered, as the lower L* and higher a* might also be related to the higher age, due to higher myoglobin content in the MPS of the LD animals. Again, this assumption was supported by several reports [41,42,43] and in agreement with color values for the older (LD) compared to younger (Ross) birds.

DL- and TL- results of the MPS were higher (*p* < 0.0001) in LD, but cooking losses of this genetic were lower (*p* = 0.0046) compared to the Ross birds (Table 2). These findings agree with other studies that also showed reduced water holding capacities in LD, as well as in other slow-growing birds [26,44,45]. The DL results might be related to the pH values, as meat with a lower pH_24 h p.m._ is accompanied by a higher DL [25,36,43,45]. This assumption was supported by Berri et al. [46] who found a negative correlation between ultimate pH and DL (r = −0.41; *p* ≤ 0.001). Additionally, the higher DL of slow-growing birds might also be caused by the larger surface area-to-volume ratio of the smaller breast fillets [36,45,47] or the age difference between LD and Ross birds [48]. The higher CL in Ross birds is likely related to the longer cooking time, but could also be a compensation of the increased DL and TL values, i.e., the higher DL and TL may have reduced final CL in LD birds.

Values for SF and SE were higher (*p* < 0.0001) in the LD MPS, which is concordant to findings of Mueller et al. [26], comparing LD and Ross and other slow-growing breeds. Reasons might be the differing liquid losses during storage, thawing and cooking (31.0% total loss for LD; 27.9% total loss for Ross,) and possibly structural differences of the MPS, due to the different slaughter ages. However, data about the impact of the slaughter age on tenderness, respectively shear force values, are inconsistent throughout the literature. Nakamura et al. [49] and Janisch et al. [28] found higher shear values for older compared to younger broiler, whereas Sonaiya et al. [50] and Krischek et al. [51] could not detect age-related differences. Connective tissue mainly influences the evaluation of texture attributes, which were performed by compression [52], like SF in this study. Collagen content varies between age and genetic breed, as higher collagen content was found in broiler chicken until three weeks of age and in layer birds up to five weeks, followed by decreasing contents until 10 and 15 weeks [53]. As LD is a cross between meat and layer lines [11], the influence of age and breed has to be considered. Higher collagen-content, higher cross linkage of the connective tissue and smaller fiber diameter were reported for older and slower growing birds [40,54,55]. In consequence, “more dense” connective tissue with more cross-linked collagen content might by responsible for higher shear force values in dual-purpose breed. However, further investigation is necessary to clarify the differences of the shear force values.

### 3.2. Objective Sensory Characteristics

**Figure 3:**
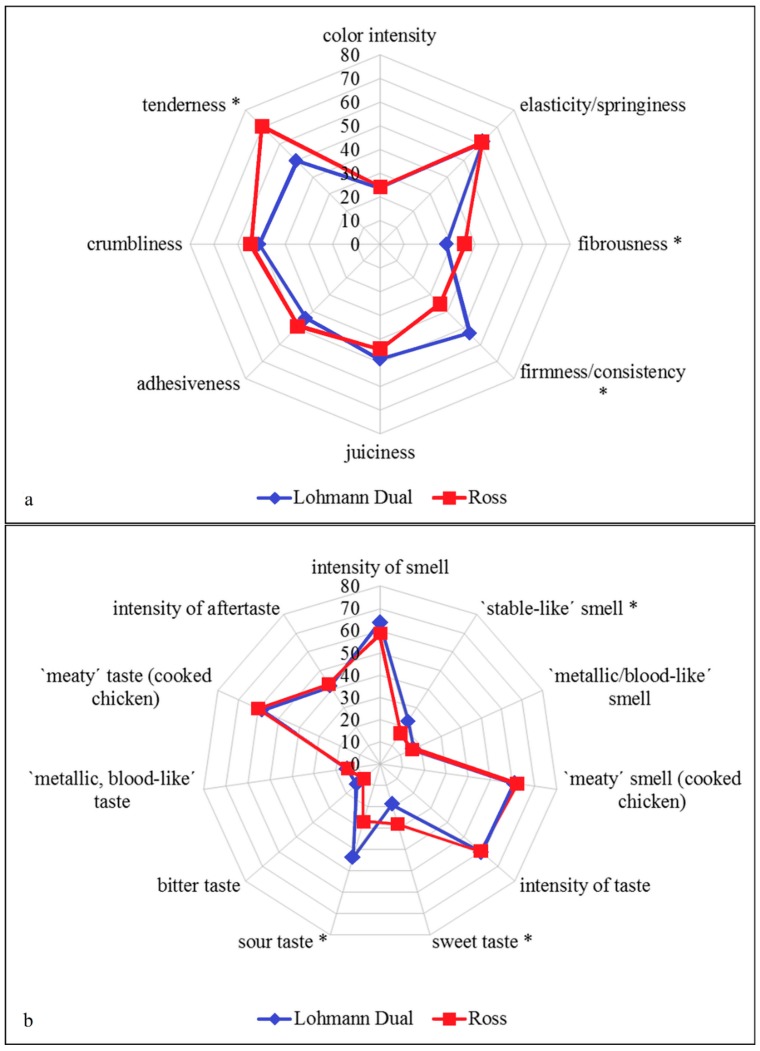
Spiderplots, with calculated means (*n* =120) for attributes smell, taste (upper figure, **a**) appearance and texture (lower figure, **b**) of the *Mm. pectorales superficiales* depending on genetic. The center represents lowest expressed values with higher ratings towards the periphery. Attributes marked with a * are significantly (*p* ≤ 0.05) different between the genetics.

Six of the 19 sensory attributes analyzed differed significantly (*p* ≤ 0.05) between the LD and Ross genetics (Table 4). For the flavor, the most distinct difference were the sour taste, which was significantly higher in LD (*p* = 0.0003) and the sweet taste that was more pronounced in Ross (*p* = 0.0053) (Figure 3a). Secondly, there were big differences in the perceived mouthfeel and texture, LD samples had firmer (*p* = 0.0005) and less tender (*p* < 0.0001) textures and tended to be juicier (*p* = 0.0501) than the Ross samples (Figure 3b). With regard to the appearance less visible fibers on the surface were detected for LD (*p* = 0.0032). Regarding the odor profile, small, but significant differences were found: Breast muscles of LD exhibited a stronger stable-like smell (*p* = 0.0349) and tendentiously a higher intensity of smell (*p* = 0.054) (Figure 3a). Appearance, texture, juiciness and flavor are important sensory quality attributes of poultry products [40,56,57] and discrimination of taste attributes is more perceptible with increasing age of birds [58].

In general, genetic variation is usually only accountable for minor differences in taste attributes [59] and the discrimination by aroma and flavor can only be performed by a subgroup of panelists, as was stated by Jahan et al. [57]. In addition to the significantly higher ‘stable-like’ smell, overall smell intensity was objectively rated tendentiously stronger in the slow-growing birds LD. These findings are appropriate to higher perceived intensities of meat odor in birds with increasing age [24,39].

The sourer perception in LD is likely related to the lower pH values [60]. The reason for the ‘sweeter’ taste of the Ross samples might be also related to this difference in pH, as less sour perception leads to a more pleasant ‘sweet’ perception. Another reason for the differing sour and sweet taste perception in the present study could be linked to differences in the muscle composition, like peptides and fat [61], as significantly differences of the fat or protein contents in the MPS of Ross and LD birds are likely present [26]. However, this would need to be further evaluated.

The higher fibrousness, meaning more visible fibers on the surface, in the Ross birds may have been caused by larger fiber swelling during cooking [62], as well as higher fiber diameters in this genetic. The latter assumption is supported by Berri et al. [48], who found larger fiber diameter in fast-growing birds compared to older intermediate and slow-growing chicken and by own unpublished results.

In this study, the LD were perceived as firmer and less tender, which was supported by numerous findings and could be also related to the significantly higher shear force and energy results in this genetic in comparison to the Ross. Lower tenderness ratings were stated for older birds [25,39,42]; as more mature tissue, due to higher collagen content, crosslinking [15,40] and thicker perimysium [42] is linked to firmer and less tender ratings. Insoluble cross-linked collagen shrinks with heating and is supposed to cause firmness and toughness, because of a smaller fiber size and greater moisture loss [15]. However; as there have been many factors, such as genotype, diet, age and deboning time, that can influence meat texture [24,63], an adjusted production line for alternative genetics, like LD, may improve texture quality characteristics. In concordance, the present results were furthermore supported by Horsted et al. [16], who found a positive correlation between tenderness and sweet taste (as was the case for the Ross samples).

Allen et al. [64] claimed negative influences of selection for higher ultimate pH and greater processing yields on sensory quality, especially juiciness. Similarly, Ross tended to be less juicy and higher juiciness was perceived in the older birds [50] of the LD genetic.

Given the results of this study, future research in sensory analysis should primarily concentrate on determining the market potential of dual-purpose chicken, primarily at the consumer level. It would be valuable to find out, how the varying sensory properties of different genetic lines agree with consumers preferences, i.e., does a target group for more firmer meat with a distinct odor and flavor profile exist? For instance, firmer meat with a more intense flavor was preferred in Thailand [35]. Furthermore, the influence of information on consumers’ acceptance, such as animal welfare throughout the production system, should not be underestimated and could influence consumer preferences.

## 4. Conclusions

The dual-purpose breed Lohmann Dual is less efficient in meat production, as lower weight properties were obtained over a longer rearing period. The sensory quality differs from the commercial Ross line; future studies should focus on the market potential of chicken meat with such flavor and texture characteristics. Eventually, sensory attributes of LD are linked to a more “traditional” eating-experience and could fit to consumers demand.

One benefit of the dual-purpose breed is the utilization of all animals either for meat or egg production, with only a few restrictions compared to highly specialized breeds. Large strides can be made in animal welfare with the production of these animals. However, each chicken breed needs an optimized husbandry system, feeding and processing requirements and further adjustments are still necessary, before dual-purpose production constitutes a reliable alternative to the culling of day-old male cockerels.

## Figures and Tables

**Table 1:** Sensory attributes and definitions for descriptive sensory analysis. This information was available for each panelist during evaluation.

Name of Attribute	Definition	How to Determine	Reference Material	Minimal and Maximal Specifications
**Dimension: Appearance**				
Color intensity	intensity of color on the surface	color intensity on the surface	bright/pale to dark	
Fibrousness	size of visible fibers on the surface	evaluation on the surface	single hair (minimum), bundles on the picture (7), rough bundles (maximum)	no visible (hair) to clearly visible fibers bundles (rough bundles)
Elasticity/springiness	ability to recover into original form after being pressed with a fork	press the entire surface with the fork	soft cheese (minimum), toast (middle), wine gum (maximum)	inelastic (shows imprints like soft cheese) to toast to highly elastic (springs back like wine gum)
**Dimension: Smell**				
Intensity of smell	overall intensity of smell	fork the sample and evaluate the smell on the bottom side		weak to very intensive
‘Stable-like’ smell	intensity of smell like dung/animals/housing		skatole, NH_3_	not perceivable to highly perceivable
‘Metallic/blood-like’ smell	intensity of smell like metal or blood		blood, iron, old money	not perceivable to highly perceivable
‘Meaty’ smell (cooked chicken)	intensity of smell like cooked meat, especially poultry meat		chicken broth, bouillon	not perceivable to highly perceivable
**Dimension: Taste and Flavor**				
Intensity of taste	overall intensity of taste	evaluation after seven to ten chews with closed mouth		weak to very intensive
Sweet taste	intensity of sweetness		2.1 g saccharose (middle); 4.2 g saccharose (maximum)	not perceivable to highly perceivable
Sour taste	intensity of sour taste		0.14 g citric acid; 0.28 g citric (high, 80%)	not perceivable to highly perceivable
Bitter taste	intensity of bitterness		0.115 g caffeine (middle); 0.21 g caffeine (maximum)	not perceivable to highly perceivable
‘Metallic, blood-like’ taste	intensity of metallic or blood-like taste (fresh drip loss)		iron sulfate, blood, ‘Kräuterblut’	not perceivable to highly perceivable
‘Meaty’ taste (cooked chicken)	intensity of meaty taste (like cooked chicken)		chicken broth	not perceivable to highly perceivable
**Dimension: Aftertaste**				
Intensity of aftertaste	allover-intensity of aftertaste	evaluation 5 s after swallowing		not perceivable to highly perceivable
**Dimension: Texture**				
Firmness/consistency	power, to divide the sample with the incisors	use your incisors and start biting on the surface to bite a 1 cm-piece off	soft cheese (minimum), Gouda cheese (middle), carrot/Werthers Original Bonbon (maximum)	soft (incisors cut easily through the sample) to firm (sample crushes)
Juiciness	amount of juice while chewing	chewing with the molars for two to three times	Banana (minimum), cucumber (middle), mandarin (maximum)	not juicy to highly juicy
Adhesiveness	coherence while chewing and adhesion to the molars	chewing with the molars for two to three times	cucumber (minimum), gouda cheese (middle), toffee-bonbon/dry fruit (maximum)	not adhesive to highly adhesive
Crumbliness	amount of particles existing before swallowing and rate for coherence	evaluation of the particles/fibers	mash (minimum), gouda cheese (middle), shortbread (maximum)	not crumbly to crumbly
Tenderness	power needed to chew	chewing until swallowing	jerky meat (minimum), seasoned apricot (maximum	chewy to tender

**Table 2:** Mean, standard deviation (SD) and degrees of freedom (df) of carcass-characteristics for Ross and Lohmann Dual.

	Ross (*n* = 30)		Lohmann Dual (*n* = 30)		df	*p*-Values
	Mean	SD	Mean	SD		
Carcass weight (g)	2182.5	131.3	1415.7	126.7	58.0	<0.0001
Breast weight (g)	549.1	50.2	179.6	20.6	38.6	<0.0001
Leg weight (g)	596.9	38.6	440.9	41.3	50.1	<0.0001
Breast yield (%)	25.1	1.3	12.7	0.8	58.0	<0.0001
Leg yield (%)	27.4	1.2	31.2	1.1	58.0	<0.0001

Weights were determined 24 h after slaughter (p.m.). Breast and leg yields are related to the carcass weight.

**Table 3:** Mean and standard deviation (SD) of *Musculus pectoralis superficialis* meat quality traits.

	Ross (*n* = 30)		Lohmann Dual (*n* = 30)		df	*p*-Values
	Mean	SD	Mean	SD		
pH 24 h p.m. *	5.8	0.2	5.7	0.3	32.8	0.0079
L* 24 h p.m.	61.1	2.1	57.0	2.9	58.0	<0.0001
a* 24 h p.m. *	1.9	0.8	3.1	1.1	58.0	<0.0001
b* 24 h p.m.	5.1	1.4	1.9	1.1	58.0	<0.0001
Drip loss, DL (%)	0.6	0.3	1.3	0.5	44.4	<0.0001
Thawing loss, TL (%)	2.2	1.0	6.9	1.7	47.0	<0.0001
Cooking loss, CL (%) *	25.0	3.0	22.8	2.8	58.0	0.0045
Shear force, SF (N)*	5.2	0.9	7.1	1.1	58.0	<0.0001
Shear energy, SF (N x mm)	11.2	2.3	15.7	3.8	48.1	<0.0001

Lightness (L*), redness (a*) and yellowness (b*) values were determined 24 h after slaughter (24 h p.m.), drip loss was determined between 24 h and 72 h p.m. SF, shear force; SE, shear energy.

**Table 4:** Results from sensory evaluation of *Musculus pectoralis superficialis*. Means and standard deviation (SD) for all attributes defined.

	Ross (*n* = 24)		LD (*n* = 48)		df	*p*-Values
	Mean	SD	Mean	SD		
**Dimension: Appearance**						
Color intensity	24.0	13.5	24.0	13.3	9	0.9771
Fibrousness	35.6	22.6	28.0	18.4	9	0.0032
Elasticity/springiness	60.6	19.2	61.3	18.2	9	0.6624
**Dimension: Smell**						
Intensity of smell	58.7	17.1	63.7	12.9	9	0.0540
‘Stable-like’ smell	16.8	11.9	23.2	18.2	9	0.0349
‘Metallic/blood-like’ smell	15.5	9.6	16.6	10.6	9	0.3172
‘Meaty’ smell (cooked chicken)	62.4	12.6	61.0	11.3	9	0.3682
**Dimension: Taste**						
Intensity of taste	59.6	13.1	60.4	11.8	9	0.6919
Sweet taste	28.2	17.4	18.9	17.8	9	0.0053
Sour taste	26.8	18.2	43.6	21.7	9	0.0003
Bitter taste	9.9	8.6	13.9	13.1	9	0.0844
‘Metallic/blood-like’ taste	14.6	9.2	14.9	10.0	9	0.8452
‘Meaty’ taste (cooked chicken)	60.3	13.7	58.4	12.5	9	0.3298
**Dimension: Aftertaste**						
Intensity of aftertaste	42.7	16.8	41.5	17.6	9	0.3755
**Dimension: Texture**						
Firmness/consistency	35.8	17.0	53.2	15.7	9	0.0005
Juiciness	44.4	14.0	48.6	14.7	9	0.0501
Adhesiveness	48.8	19.8	44.2	19.4	9	0.2918
Crumbliness	54.4	16.8	50.9	17.5	9	0.1089
Tenderness	70.2	14.6	49.7	19.8	9	<0.0001

Values representing ratings on an unstructured 100-point-scale from 0 (the weakest perception) to 100 (the strongest perception) without units.
